# Design and Fabrication of High-Efficiency, Low-Power, and Low-Leakage Si-Avalanche Photodiodes for Low-Light Sensing

**DOI:** 10.1021/acsphotonics.3c00026

**Published:** 2023-05-04

**Authors:** Amita Rawat, Ahasan Ahamed, Cesar Bartolo-Perez, Ahmed S. Mayet, Lisa N. McPhillips, M. Saif Islam

**Affiliations:** Electrical and Computer Engineering, University of California − Davis, Davis, California 95616, United States

**Keywords:** avalanche photodiodes, photon-trapping microholes, Si-APD, short-infrared wavelength, visible wavelength

## Abstract

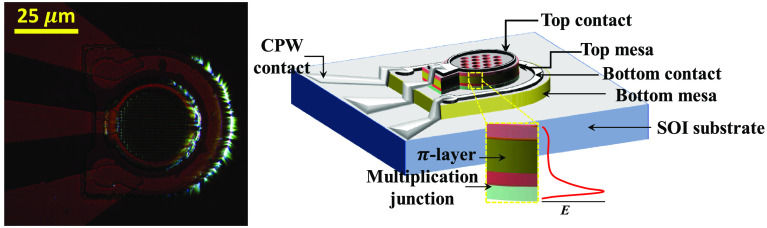

Since the advent of impact ionization and its application in avalanche photodiodes (APD), numerous application goals have contributed to steady improvements over several decades. The characteristic high operating voltages and the need for thick absorber layers (π-layers) in the Si-APDs pose complicated design and operational challenges in complementary metal oxide semiconductor integration of APDs. In this work, we have designed a sub-10 V operable Si-APD and epitaxially grown the stack on a semiconductor-on-insulator substrate with a submicron thin π-layer, and we fabricated the devices with integrated photon-trapping microholes (PTMH) to enhance photon absorption. The fabricated APDs show a substantially low prebreakdown leakage current density of ∼50 nA/mm^2^. The devices exhibit a consistent ∼8.0 V breakdown voltage with a multiplication gain of 296.2 under 850 nm illumination wavelength. We report a ∼5× increase in the EQE at 850 nm by introducing the PTMH into the device. The enhancement in the EQE is evenly distributed across the entire wavelength range (640–1100 nm). The EQE of the devices without PTMH (flat devices) undergo a notable oscillation caused by the resonance at specific wavelengths and show a strong dependency on the angle of incidence. This characteristic dependency is significantly circumvented by introducing the PTMH into the APD. The devices exhibit a significantly low off-state power consumption of 0.41 μW/mm^2^ and stand fairly well against the state-of-the-art literature. Such high efficiency, low leakage, low breakdown voltage, and extremely low-power Si-APD can be easily incorporated into the existing CMOS foundry line and enable on-chip, high-speed, and low-photon count detection on a large scale.

## Introduction

High-speed and efficient detection of visible and near-infrared (IR) electromagnetic (EM) wavelengths is essential for ceaselessly unfolding novel imaging and communication applications, including data and telecommunication, augmented reality/virtual reality (AR/VR),^1,2^ light detection and ranging (LiDAR),^3^ visible light communication (VLC),^4,5^ near-IR/thermal imaging, fluorescent lifetime imaging microscopy (FLIM),^6^ and internet-of-things.^7^ Group III–V compound semiconductor-based detectors are most widely used due to their direct bandgap and high carrier mobility.^8^ However, the incompatibility with the complementary metal gate oxide semiconductor (CMOS) process line results in a tedious and expensive 3D heterogeneous integration.^9^ Silicon photonics is one of the rapidly growing fields due to its CMOS compatibility and high-volume manufacturability resulting in a high data communication rate enabled by monolithic integration of optical and logic circuitry.^10^ Several detectors including metal–semiconductor-metal (MSM)^11−13^ and PIN photodiodes^14−16^ have been thoroughly explored to enable visible and near-IR wavelength detection utilizing group-IV semiconductors. In the MSM and PIN devices, photon detection efficiency solely depends on the intrinsic absorption limit, and bandgap-limited generation and recombination rate of silicon. Therefore, a high detection efficiency demands a thick absorber layer which leads to a high parasitic resistance (*R*) due to a longer carrier transportation path. High detection efficiency is vital for advanced optoelectronic applications, especially in low-light conditions. The devices without any intrinsic gain mechanism, are incapable of catering to such pivotal photon detection needs. The photomultiplier tubes^17^ were used earlier to efficiently detect low-photon count, although the size of the device makes it impractical to embed on-board let alone on-chip. Since the advent of impact-ionization-based avalanche photodiodes (APDs),^18−23^ there has been an accelerated growth toward integrating high-speed optics on-board and on-chip. Despite significant development in the design optimization of Si-APD, the high operating voltage, low intrinsic EM wave absorption in Si, and high dark current remain challenging.^24−31^ To enhance the detection efficiency, numerous methods such as metal-grating,^32,33^ photon-trapping holes,^14,28,29^ antireflection coating,^34^ and so on have been extensively explored.

In this work, at first, we designed a sub-10 V Si-APD device using the Silvaco Atlas TCAD simulation platform. In alignment with the designed doping profile, we epitaxially grow the APD stack on a silicon-on-insulator (SOI) wafer and fabricated the Si-APD device structures. To enhance the absorption efficiency in the device, we introduced photon-trapping microholes (PTMH) into the device. Finally, we present a detailed direct-current (DC) current–voltage (*I*–*V*) characterization of the devices for a wide range of illumination wavelengths. We show an exceptionally low off-state current and a considerable increase in the quantum efficiency of the device by introducing the photon-trapping holes. We also show a drastic change in the multiplication gain with the illuminated laser power. Further, we show that without PTMH, devices (flat devices) are sensitive to the illumination direction, which is significantly mitigated by introducing the PTMH. A low reverse-biased current, a sub-10 V breakdown voltage, enhanced wavelength absorption, and CMOS compatibility of these Si-APDs have the potential to detect ultralow photon counts and can leapfrog the on-chip integration of the photonic devices.

## Methods

**Figure 1:**
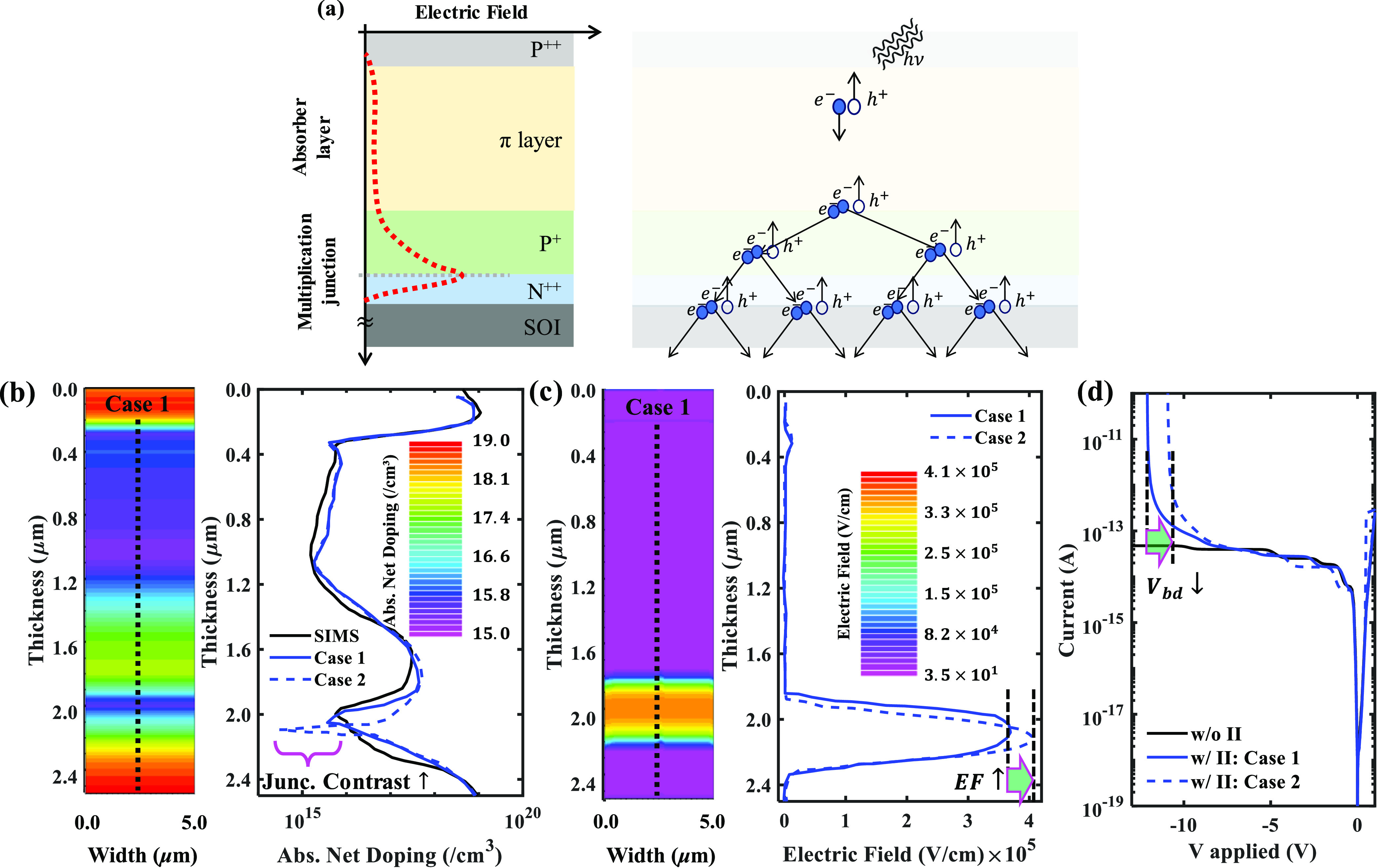
(a) Schematic of the doping stack, an electric field profile (red dotted curve), and an illustration of the avalanche phenomenon triggered by impact ionization in avalanche photodiodes. (b) 2D contour plot of the doping profile for the case 1 scenario used in Si-APD structure simulated in Silvaco Atlas TCAD and a 1D doping profile extracted at the black dotted cutline. In the 1D plot, the case 1 doping scenario is compared against the case 2 doping variant and the SIMS doping profile of the epitaxially grown APD stack used for the APD fabrication. The doping contrast at the multiplication junction is engineered to trigger an early impact ionization followed by an early avalanche breakdown. (c) A 2D contour plot of the electric field (EF) profile in case 1 Si-APD, and a 1D EF profile extracted at the black dotted cutline. The EF profile in the case 1 doping scenario is compared against that of case 2 to show an increase in the EF with an increase in the doping contract at the multiplication junction. (d) Current–voltage profile of the Si-APD is simulated in Silvaco to capture the avalanche breakdown. An increase in the electric field at the multiplication junction results in a reduced breakdown voltage.

The doping profile of a reach-through APD consists of a p++ (n++) contact layer followed by an intrinsic π-layer and a p+/n++ (n+/p++) multiplication junction, as shown in Figure 1a. The thickness and the bandgap of the π-layer govern the fundamental limit of photon absorption efficiency in the device and the parasitics. The doping contrast at the p+/n++ multiplication junction governs the breakdown voltage of the APD. An illustration of the *e*^–^–*h*^+^ pair generation due to the illumination and multiplication of the generated carriers due to the high electric field is shown in Figure 1a.

We engineered the APD doping profile to manipulate the electric field profile at the multiplication junction to trigger a low-voltage impact ionization (II) (i.e., a low-voltage avalanche breakdown). The electric field (EF) required for Si to break down is ∼3 × 10^5^V/cm.^18,35^ We simulate two doping variants for the APD (cases 1 and 2) to compare the EF profile at the multiplication junction. In Figure 1b, we show a 2D contour plot and a 1D cutline (extracted at the black dotted line) comparing the doping profiles in both case 1 and case 2 APDs. The doping contrast at the multiplication junction of case 2 APD is sharper as compared to case 1 APD. A 2D contour plot of the EF profile in case 1 and a comparison of the EF profiles in both cases are shown in Figure 1c. An increased doping contrast in case 2 results in an increased EF at the multiplication junction. This increased EF translates to an early trigger of the II and avalanche breakdown. Finally, we have generated *I*–*V* profiles in both cases by enabling (blue trend) and disabling (black trend) the II physics model in the simulation framework. A direct correlation between increased EF and reduced breakdown voltage is shown in Figure 1d. It is to be noted that, the purpose of the simulation exercise is to estimate the breakdown voltage. Therefore, we have simulated the APD device structures under ideal conditions (i.e., there are no surface state traps present).

We epitaxially grew the APD stack on an SOI wafer in alignment with the optimized doping profile. A secondary ion mass spectrometry (SIMS) imaging of the epitaxially grown stack showcasing the doping profile of the wafer is presented in Figure 1b (black trend).

**Figure 2:**
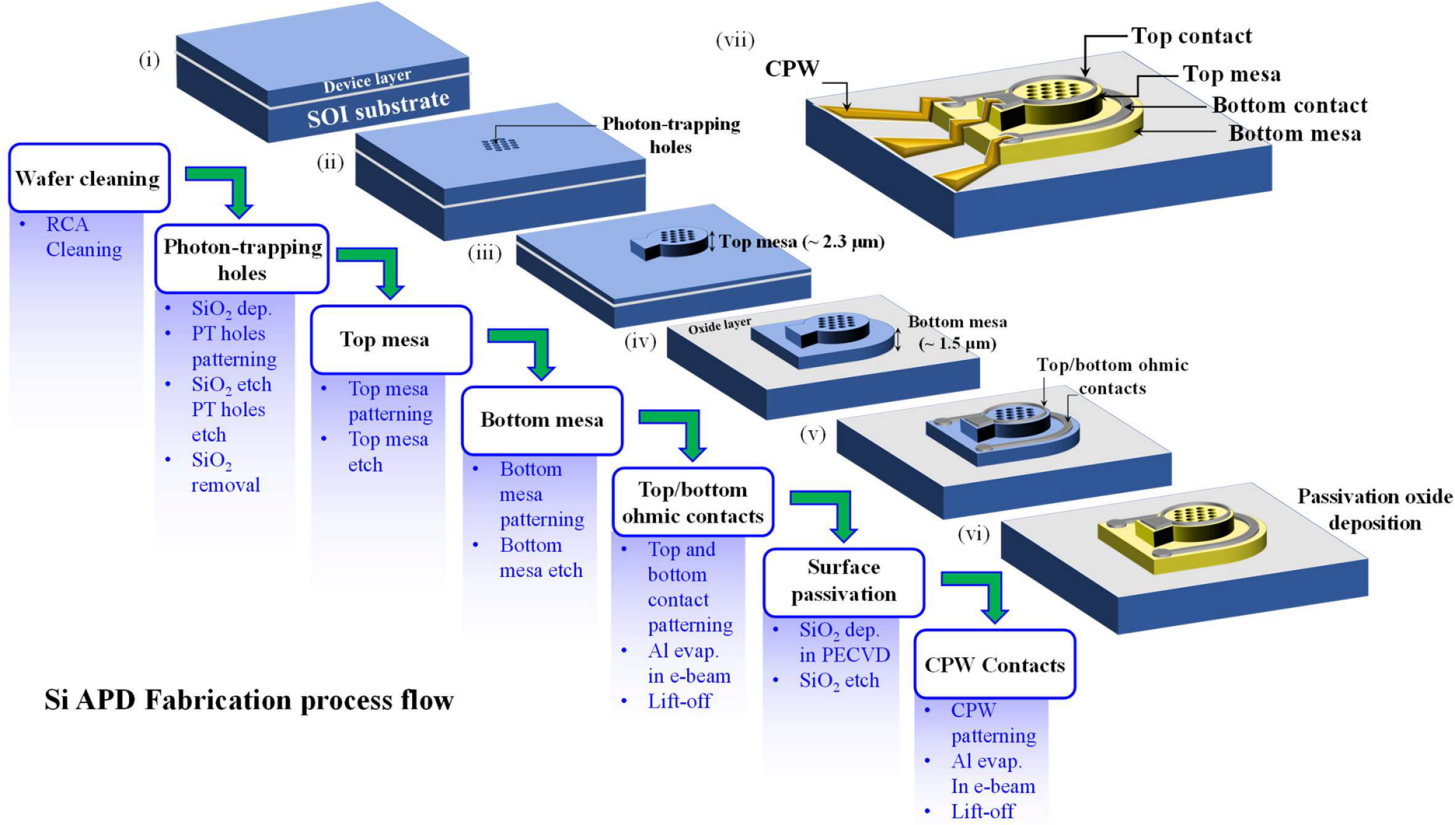
Flowchart briefly describing the CMOS-compatible processes used to fabricate Si-APDs. The photon-trapping microholes (PTMH) are patterned using a stepper optical lithography system. The mesa and the PTMH surfaces are passivated using SiO_2_ coating to reduce the off-state leakage current. To reduce contact resistance and enable high-speed operations, we have incorporated coplanar-waveguide (CPW) contacts.

We start the fabrication by cleaning the wafer using the RCA cleaning process, a standard Si wafer cleaning process (step (i) in Figure 2). Right after cleaning, we deposit a 200 nm thick SiO_2_ layer and pattern the photon trapping microhole (PTMH) array using a UV lithography in a stepper system. Next, the PTMH patterns are transferred on the SiO_2_ layer using an inductively coupled plasma reactive ion etching (ICPRIE) process. Using the etched SiO_2_ as a hard mask, the PTMH array is transferred on the APD wafer using the ICPRIE process (step (ii) in Figure 2). Next, we etch the top mesa in alignment with the PTMH array to expose the bottom highly doped n++ contact layer followed by the bottom mesa etching to isolate the devices using the ICPRIE process (step (iii-iv) in Figure 2). Next, we pattern the top and the bottom contact using E-beam evaporation of aluminum (step (v) in Figure 2). Next, we passivate the side walls of the hole array and the mesas by depositing SiO_2_ in plasma enhanced chemical vapor deposition (PECVD) system. Finally, we selectively etch the SiO_2_ layer from the top of the device and pattern the coplanar waveguide (CPW) contacts (step (vi-vii) in Figure 2). All the processes used are standard CMOS-compatible processes. We have performed a detailed DC I–V characterization of the fabricated devices using a Semiconductor Device Parameter analyzer and NKT Photonics supercontinuum Laser source interfaced and controlled by a LabVIEW framework. The systemic noise in the parameter analyzer is of the order of 10 pA. A tunable laser NTK source with wavelength turnability between 640 and 1100 nm is used to illuminate the APDs.

## Results and Discussion

**Figure 3:**
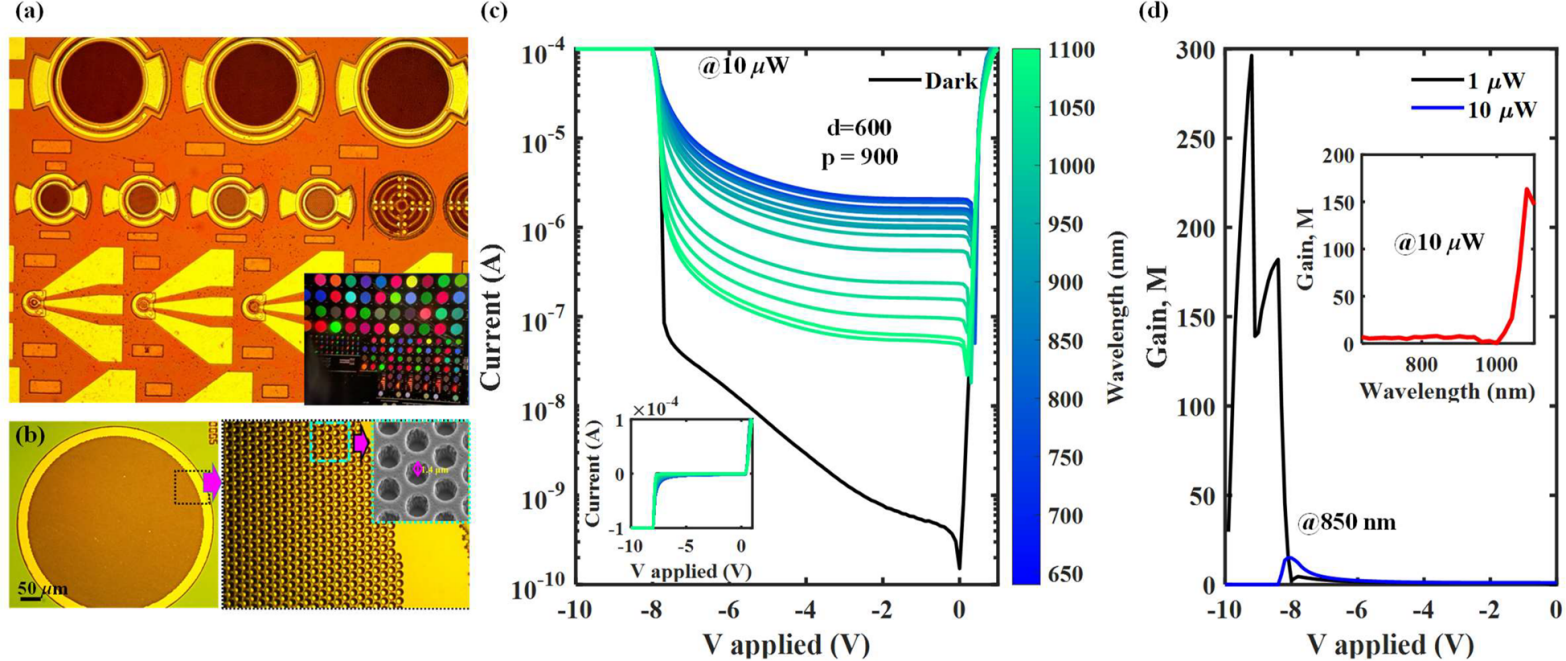
(a) Micrograph of the Si-APD devices fabricated and the inset shows a microscopic image captured while illuminating the die with white light. A spectrum of wavelength reflected from a variety of devices with varying PTMH diameter (*d*) and periodicity (*p*) evincing the wavelength selectivity of the devices. (b) A microscopic image showcasing the hexagonal lattice arrangement of the PTMH array introduced in the Si-APD device and a 150× enlargement of the PTMH features. The inset shows the SEM profile of the PTMH array highlighting the depth and the sidewall surface profile of the PTMH. (c) The dark and illumination DC *I*–*V* characteristics measured (at a fixed laser power = 10 μW) for with-PTMH Si-APD (*d* = 600 nm; *p* = 900 nm) are plotted on a semilog scale. The inset shows the *I*–*V* trends on a linear scale. A gradual reduction in the illumination current with the increase in the wavelength reflects the fundamental absorption characteristics of Si. (d) The multiplication gain in the device extracted at −1 V unity gain voltage. The gain curve is compared for 1 and 10 μW laser power of 850 nm illumination wavelength. The multiplication gain increases at lower illumination power due to a reduced carrier–carrier scattering. The inset in (d) shows the *M* extracted from (c) as a function of illumination wavelength. A rapid increase in the gain at longer wavelengths is attributed to reduced carrier–carrier scattering due to low carrier generation (PTMH: photon-trapping microhole).

The optical micrograph of the fabricated devices is shown in Figure 3a. The die consists of devices with diameters varying from 25 to 500 μm. The diameter (*d*) and the periodicity (*p*) of the PTMH vary from 600–1500 nm and 900–3000 nm, respectively. The inset of Figure 3a shows a microscopic image of the devices with varying PTMH diameter and periodicity, selectively reflecting certain wavelengths from the microscopic white light. This preferential reflection of certain wavelengths from the devices has the potential to devise an on-chip spectrometer.^36^Figure 3b shows an enlarged 500 μm size device with PTMH, a 150× magnified microscopic image, and the inset showing a scanning electron microscopic (SEM) image of the PTMH array. We characterize the APD for an applied bias range varying from 0 to 1 V in the forward bias and 0–10 V in the reverse bias mode. The optoelectronic characteristics are studied for a wavelength range varying from 640 to 1100 nm.

### DC *I*–*V* Characteristics

**Figure 4:**
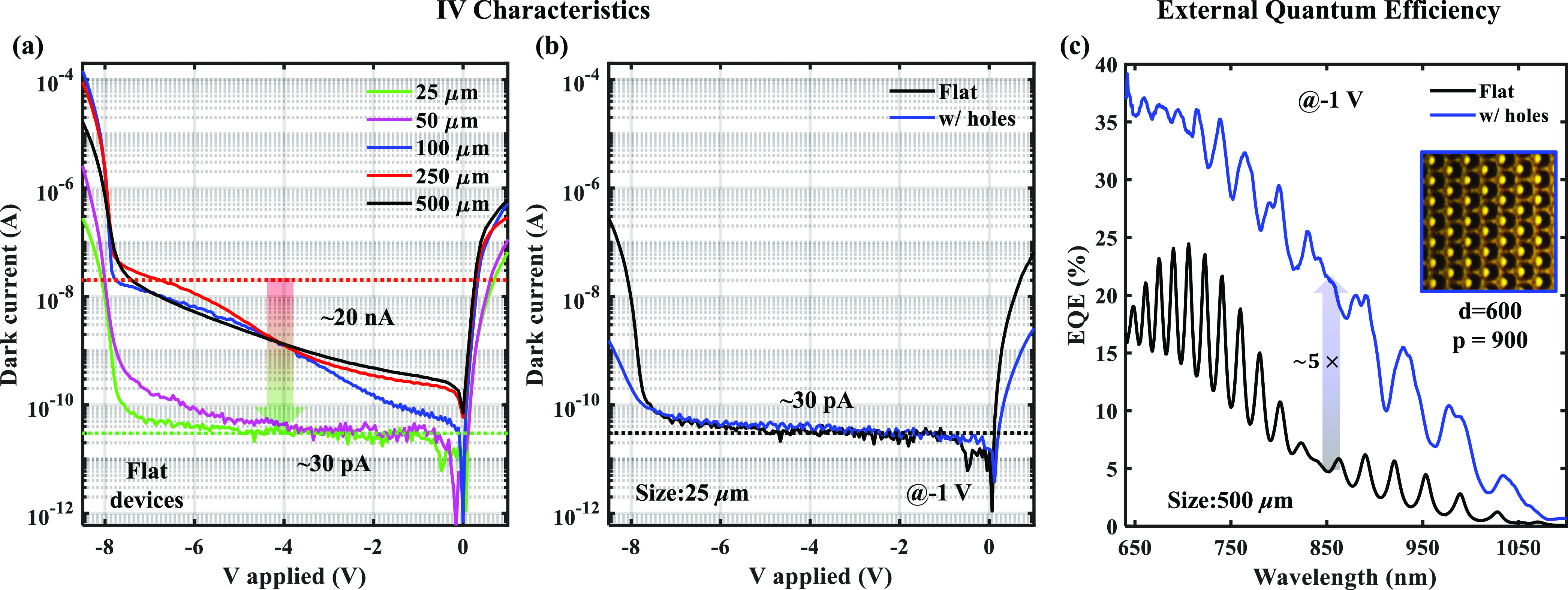
(a) Dark current of the flat APDs with device diameter scaling. The dark current scales aptly with the device size. (b) Impact of PTMH on the dark current of the 25 μW device diameter. The SiO_2_-based dangling bond passivation reduces the surface state and results in comparable dark currents in both flat and with-PTMH devices. (c) External quantum efficiency trends as a function of illumination wavelength. The EQE shows ∼5× enhancement by introducing the PTMH structures into the flat device.

In Figure 3c, we have plotted the DC *I*–*V* characteristics under dark and illumination conditions for a with-PTMH device (device diameter = 500 μm; PTMH diameter = 600 nm; periodicity = 900 nm) on a semilog scale. A high current in the forward bias represents a conventional on-state diode characteristic. A low reverse-biased current and an eventual impact-ionization-induced breakdown represent the APD characteristics. The inset of the figure plots a linear scale *I*–*V* profile in the dark and under illumination. The wavelength sweep varies from 640 to 1100 nm. The laser power is maintained at 10 μW during the entire wavelength sweep. A gradual decrease in the current with an increase in the wavelength is accredited to the reduced absorption coefficient at longer wavelengths in silicon. Aligned with the expectation, the Si-APD shows a sub-10 V breakdown (∼8.0 V). In Figure 3d, we present the multiplication gain (*M*) of the APDs for 1 and 10 μW laser power for the 850 nm illumination wavelength. The multiplication gain is calculated at the unity gain voltage (*V*_*M*=1_ = −1 V) and using *M* = (*I*_photo_ – *I*_dark_)/(*I*_photo_(*V*_*M*=1_) – *I*_dark_(*V*_*M*=1_)) expression.^29^ The gain, *M*, increases rigorously with reduced laser power. An enormous amount of *e*^–^–*h*^+^ pair generation at a high laser power causes excessive carrier–carrier scattering that results in a compromised carrier multiplication. A significant increase in the *M* at low laser power makes these APDs suitable for low-photon detection applications. The inset of Figure 3d shows the gain versus wavelength trend calculated from Figure 3c. A reduced carrier–carrier scattering resulting from a reduced generation has resulted in an enormous increase in the gain at longer wavelengths. Further, in Figure 4a, we have compared the dark state current of flat devices with device diameter scaling from 500 to 25 μm. The dark current at prebreakdown bias scales from 20 nA to 30 pA with the diameter scaling. The 25 and 50 μm diameter devices show dark currents as low as ∼30 pA, which is expected to reduce further as the dark current reaches the systemic noise floor. Finally, we have compared the impact of PTMH on the dark current in Figure 4b. Due to surface passivation, the impact of PTMH-induced surface states is negligible on the dark current, and an equivalent dark current of ∼30 pA is achieved even after introducing the PTMH array.

### EQE: With and Without PTMH

We show a comparison of the external quantum efficiency (EQE) for a without-PTMH device (flat device) against the device with-PTMH in Figure 4c. We have considered a hexagonal lattice arrangement for the PTMH array (PTMH diameter = 600 nm; periodicity = 900 nm), as shown in the inset of Figure 4c. The flat device shows a maximum of 25% EQE (black curve) at 700 nm wavelength and drops rapidly at longer wavelengths. We observe an oscillatory pattern in the EQE of the flat device. The oscillation EQE is due to the resonance of certain wavelengths in the Si active layer sandwiched between the air and the SiO_2_ as air/Si/SiO_2_, where the SiO_2_ layer acts as a back reflector.^37−39^ As the refractive index of Si (*n*_Si_) is greater than that of the air (*n*_air_) and the SiO_2_, i.e., , which causes the cavity effect.^37,39^ Due to this cavity effect, certain wavelengths get trapped in the Si and result in enhanced EQE. Thereafter, we show an ∼5× enhancement in the EQE at 850 nm with the introduction of PTMH. The PTMH works as a waveguide and allows lateral propagation by bending the light^14,28,29^ almost 90°. This bending can be attributed to the diffraction of the incident light at the corners and edges of the PTMH as the feature size of the holes is comparable to that of the illumination wavelength.^40^ Lateral propagation of the incident light increases the path length of the light inside the absorber layer, which therefore increases the absorption.
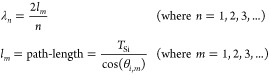
1

### EQE: Incidence Angle Dependency

**Figure 5:**
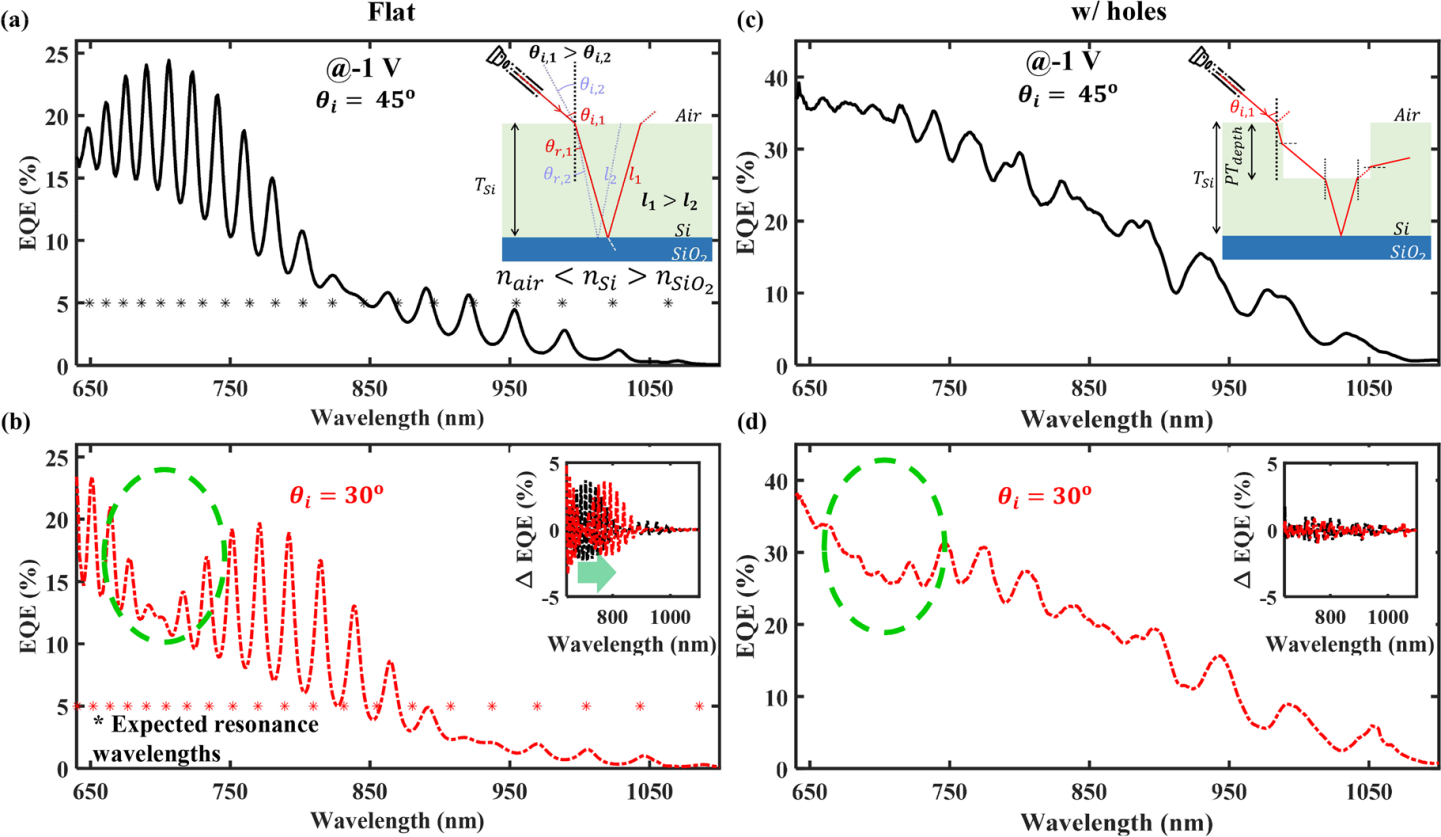
EQE profile as a function of wavelength captured at 45° and 30° angles of incidences of the laser used to illuminate the flat and with-PTMH devices. (a, b) Oscillatory EQE profiles captured for the flat device and the asterisks in (a) and (b), marking the possible resonance wavelengths mathematically calculated for 45° and 30° incidence angles. The inset of (a) shows a schematic of the EM wave refraction while entering the Si region bounded by air and SiO_2_ at two different angles of incidences (θ_*i*,1_ > θ_*i*,2_) to show path-length modulation (*l*_1_ > *l*_2_). This path-length modulation leads to a systematic shift in the oscillatory ΔEQE profile, as shown in the inset of (b). The EQE of the device with-PTMH at (c) 45° and (d) 30° angles of incidences. The presence of PTMH perturbs the smooth resonance process as shown in the inset of (c) and results in the dilution of oscillations and prominent incidence angle dependency as highlighted in the inset of (d). The drop in the EQE near the 700 nm wavelength range in (d) marked with a green dotted line is a residual effect of the prominent drop present near the 700 nm wavelength range in (b) marked with a green dotted line.

Lastly, we have investigated the angle of incidence sensitivity on the EQE. We measured the EQE of a flat device at 45° and 30° angles of incidence. Figure 5a,b shows EQE trends for a flat device. We observe prominent oscillation in both cases due to the resonance at certain wavelengths. The wavelengths that satisfy eq 1 result in a standing wave formation, i.e., they undergo resonance.^41^ Inset of Figure 5a demonstrates the path-length change from *l*_1_ to *l*_2_ for θ_*i*,1_ and θ_*i*,2_ angles of incidence. The estimated wavelength points expected to show resonance are calculated using eq 1 and are plotted in Figure 5a,b using asterisks (*). The inset of Figure 5b plots the ΔEQE, highlighting a systematic right shift in the oscillating EQE profile with an angles of incidence change from 45° to 30°. Figure 5c,d show the EQE profile of a with-PTMH device with 45° and 30° angles of incidences. The systematic shift in the oscillating EQE profile present in the flat device has been diluted with the introduction of PTMH. The presence of PTMH creates a perturbation in the EM wave travel path and disrupts the resonance phenomenon as shown in the inset of Figure 5c. The ΔEQE profiles at both 45° and 30° angles of incidences for the with-PTMH device are shown in the inset of Figure 5d. The prominent shift evident in the flat device’s EQE is indistinguishable in the with-PTMH device. A significant drop in the EQE in a flat device for a 30° angle of incidence near the 700 nm wavelength (marked with a green dotted line in Figure 5b) has a residual effect on the EQE of the with-PTMH device as well (marked in Figure 5d with a green dotted line). Adding PTMH interrupts the resonance phenomenon and resolves the incidence angle dependency of the EQE profile. This dependency can be further reduced by changing the PTMH etch profile from cylindrical to tapered.^14^

### Device Performance Benchmarking

Finally, we present a device performance benchmarking against state-of-the-art literature as listed chronologically in Table 1. The off-state dark current density measured right before the breakdown voltage compares fairly well against the literature. The presented devices show an avalanche breakdown at ∼8.0 V. The introduction of the PTMH array results in 5× enhancement in the EQE superseding the fundamental absorption limit of 0.8 μm thick π-layer (calculated as ∼5%^42^). The EQE of the device is compared against the literature at a fixed illumination wavelength of 850 nm. A proportionate reduction in the EQE from 39%^29^ to 22.5% is due to a significant reduction in the π-layer thickness from 2.0 to 0.8 μm. Finally, we have compared the multiplication gain (*M*) of the device. The multiplication gain is comparable to that of ref (29) at 850 nm wavelength, whereas the *P*_off_ shows a tremendous reduction from 2.8 to 0.41 μW/mm^2^. Finally, we have estimated the excess noise factor (*F*) of the device using McINTYRE’s model.^43,44^ The calculated *F* is comparable to the literature. A detailed *F* calculation using the depletion capacitance, and the capacitance–voltage behavior of the device is presented in the supplementary document. The overall device performance shows notable improvement in comparison to state-of-the-art literature.

**Table 1:** Benchmarking Table Comparing the Device Performance against Existing Literature

					EQE(%) @850 nm			
ref	π-layer (μm)	*I*_off_ (nA/mm^2^) @prebreakdown	*V*_bd_ (V)	*P*_off_ (μW/mm^2^)	flat	w/PTMH	gain, *M*	noise factor, *F*
2007^24^	1.00	11.50	10.8	0.12	8.0		3.0 @850 nm	2.5
2014^25^	1.00	39.80	14.0	0.56	<1.0			
2017^26^	0.70	1.02 × 10^4^	10.8	110.20			5.0 × 10^3^ @280 nm	
2020^27^	0.64	730.50	8.6	6.30	<20.0		8.0 @386 nm	
2022^29^	2.00	1.27 × 10^3^	22.0	2.80	14.0	39.0	515.8 @850 nm	
comm.^30^	NA	0.15	300.0	0.05	60.0		100 @800 nm	0.3
this work	0.80	50.90	8.0	0.41	4.5	22.5	296.2 @850 nm	1.8

## Conclusion

We present a method to design and fabricate a sub-10 V Si-APD. We engineer the doping profile to achieve the desired electric field in the APD to enable a sub-10 V breakdown using the Silvaco atlas TCAD simulator. In alignment with the optimized doping profile, we epitaxially grow the APD stack and fabricated the Si-APDs. Further, the photon-trapping micro hole (PTMH) array is introduced into the devices to enhance absorption efficiency. The devices show ∼8.0 V breakdown voltage as per the design, and an exceptionally low dark current of ∼30 pA for a device with a 25 μm diameter even with the incorporation of PTMH structures. The APD shows a high multiplication gain, *M* of 296.2 at 850 nm with 10 μW laser power. Introducing the PTMH results in enhanced absorption and increases the EQE by ∼5× at 850 nm. The enhancement is uniformly distributed across the wavelength range varying from 640–1100 nm. Due to a smooth SOI interface, we show a prominent resonance occurring at certain wavelengths and causing an oscillatory EQE profile for the flat device. We also show a strong dependency on the illumination direction of the EQE profile in the flat device. This prominent EQE modulation is circumvented by introducing the PTMH array into the APD structure. The fabricated devices show excellent sensitivity, gain, and dark current performance, surpassing most of the performance parameters reported in the state-of-the-art literature.

## Supporting Information Available

Scanning electron microscopy of PTMH; Capacitance-voltage measurement; Excess noise factor calculation; Supporting references; Supporting figures (PDF)
Supporting figures (ZIP)
