# Supplementary material for: Design and Fabrication of High-Efficiency, Low-Power, and Low-Leakage Si-Avalanche Photodiodes for Low-Light Sensing
Source: ACS Photonics. 2023 May 4;10(5):1416–23. doi: 10.1021/acsphotonics.3c00026 (PMC10201457; doi:10.1021/acsphotonics.3c00026)
Supplement: Supplementary file 1 — ph3c00026_si_001.pdf [file ph3c00026_si_001.pdf]

Supplementary document
Design and fabrication of high-efficiency, low-power, and low-leakage Si-avalanche photodiodes for low-light sensing

Amita Rawat, Ahasan Ahamed, Cesar Bartolo-Perez, Ahmed S. Mayet, Lisa N. McPhillips, and M. Saif Islam*

Electrical and Computer Engineering, University of California – Davis, Davis, California 95616, USA

E-mail: sislam@ucdavis.edu

Phone: +1 (530) 754-6732. Fax: +1 530-752-8428

Scanning electron microscopy of PTMH

Figure 1 showcases the scanning electron microscopic (SEM) imaging of the Photon Trapping Micro-Holes (PTMH) with 1500 nm diameter and 2500 nm period (Fig. 1(a)). The PTMH are etched using an inductively coupled plasma reactive ion etching (ICPRIE) process and the depth is measured as 1592 nm, as shown in Fig. 1(b). The sidewall profiles are vertical due to the directionality of ICPRIE.

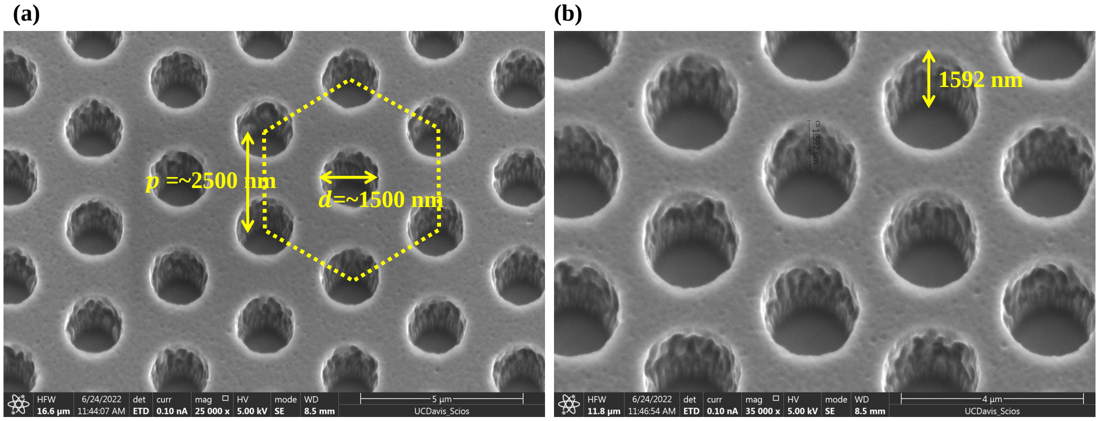

Figure 1: SEM imaging of the PTMH highlighting the (a) PT hole diameter and the period; (b) the depth of the PT hole etched using ICPRIE. The choice of ICPRIE, instead of wet etch or regular RIE has resulted in a cylindrical PTMH shape.

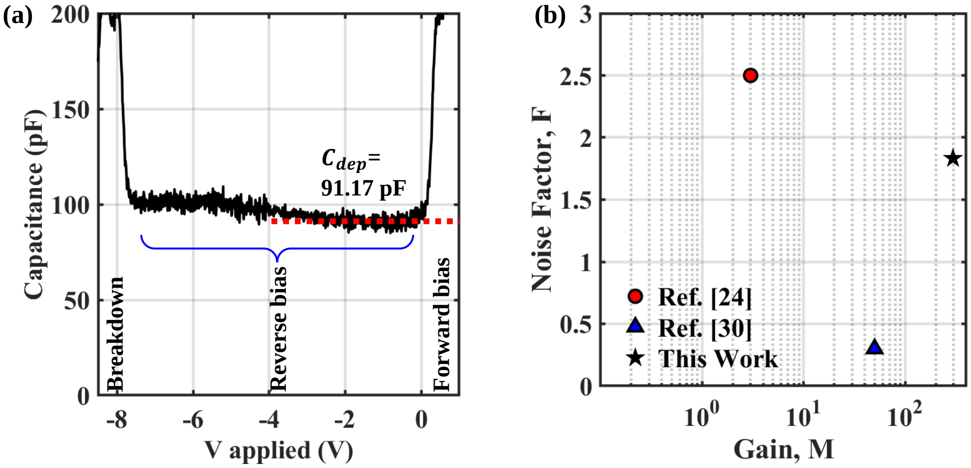

Figure 2: (a) Capacitance-voltage trend measured for a with-PTMH device ($d = 1300\ nm$; $p = 2300\ nm$) highlighting the capacitance of the device in forward, reverse, and breakdown scenarios. (b) The excess noise factor of the device is compared against the literature. The noise factor is reported to be comparable to that of the reference. [**Note:** The reference numbers are consistent with Table 1 presented in the main text.]

Capacitance-voltage measurement

The capacitance-voltage (CV) measurement of a with-PTMH device (diameter, $d = 1300$ nm and periodicity, $p = 2300$ nm) is shown in Fig. 2(a). The capacitance (C) profile is captured for 0-1 V forward bias and 0-8 V reverse bias voltages. A small alternating current (AC) signal of 50 mV RMS amplitude and 1 kHz frequency is applied to capture the carrier response. A steep increase in capacitance in forward bias is attributed to an exponential increase in conducting carriers due to the barrier lowering. The capacitance in the reverse bias is largely governed by the depletion width. The gradual increase in the capacitance that follows from the increased reverse bias is due to the minority carrier diffusion. Finally, a rapid increase in the capacitance is due to the avalanche breakdown.

Excess noise factor calculation

The excess noise factor calculation is done using McINTYRE's model.[1,2]

$$W_{dep} = \frac{A_{device} \times \epsilon_{Si}}{C_{dep}} \tag{1}$$

where W_{dep} is depletion width, C_{dep} is depletion capacitance, A_{device} is device area, and ϵ_{Si} is the electric permittivity of silicon.

$$\alpha = A \times e^{-\frac{B}{E}} \tag{2}$$

where $A = 7.03 \times 10^7\ m^{-1}$, $B = 12.30 \times 10^7\ m^{-1}$, and estimated peak electric field ($E = 4.00 \times 10^7\ V/m$).[3]

$$M = \frac{(1-k)}{e^{-(1-k)\delta} - k} \tag{3}$$

where $\delta = \alpha \times W_{dep}$.

At first, we estimated the carrier ionization ratio (k) for a given multiplication gain (M) using the depletion capacitance (C_{dep}), peak electrical field (E), and carrier ionization coefficient (α) in bulk silicon and Equations 1-3. The calculated k value for $M = 296.2$ is 1.84.

$$F = 1 - (1 - k) \times \left(\frac{M - 1}{M}\right)^2 \tag{4}$$

Using the calculated k value and respective M, the excess noise factor, F, using Equation 4.[2] A comparison of calculated F values against the literature is shown in Fig. 2(b).

References

(1) McIntyre, R. Multiplication noise in uniform avalanche diodes. *IEEE Transactions on Electron Devices* **1966**, 164–168.

(2) McIntyre, R. J. The distribution of gains in uniformly multiplying avalanche photodiodes: Theory. *IEEE Transactions on Electron Devices* **1972**, *19*, 703–713.

(3) Cheong, J. S.; Hayat, M. M.; Zhou, X.; David, J. P. Relating the experimental ionization coefficients in semiconductors to the nonlocal ionization coefficients. *IEEE Transactions on Electron Devices* **2015**, *62*, 1946–1952.
