# Supplementary figures and images for: Design and Fabrication of High-Efficiency, Low-Power, and Low-Leakage Si-Avalanche Photodiodes for Low-Light Sensing
Source: ACS Photonics. 2023 May 4;10(5):1416–23. doi: 10.1021/acsphotonics.3c00026 (PMC10201457; doi:10.1021/acsphotonics.3c00026)

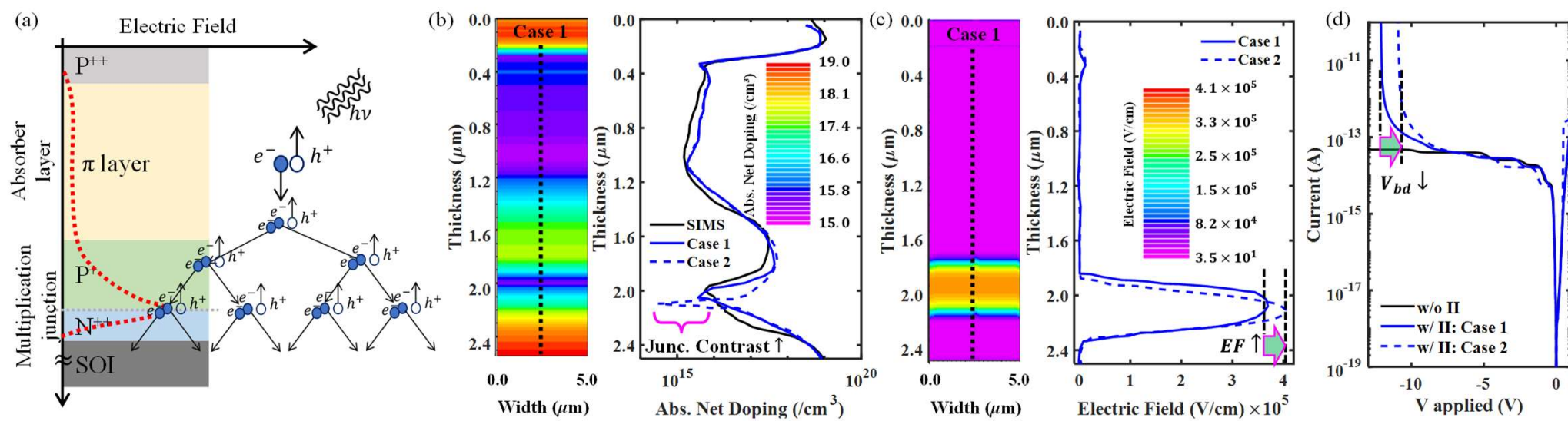

Supplement: Supplementary file 2 — ph3c00026_si_002.zip [file ph3c00026_si_002.zip › APD_Theory.pdf]

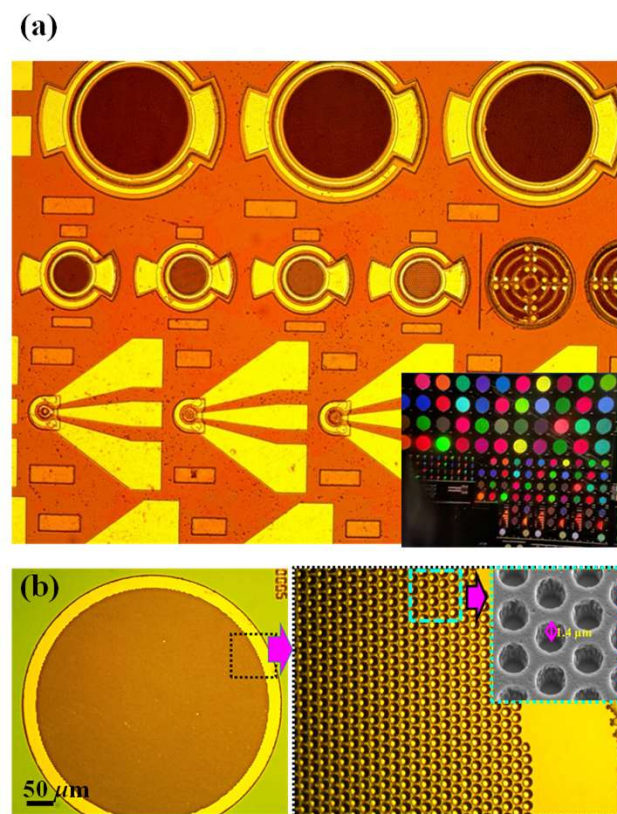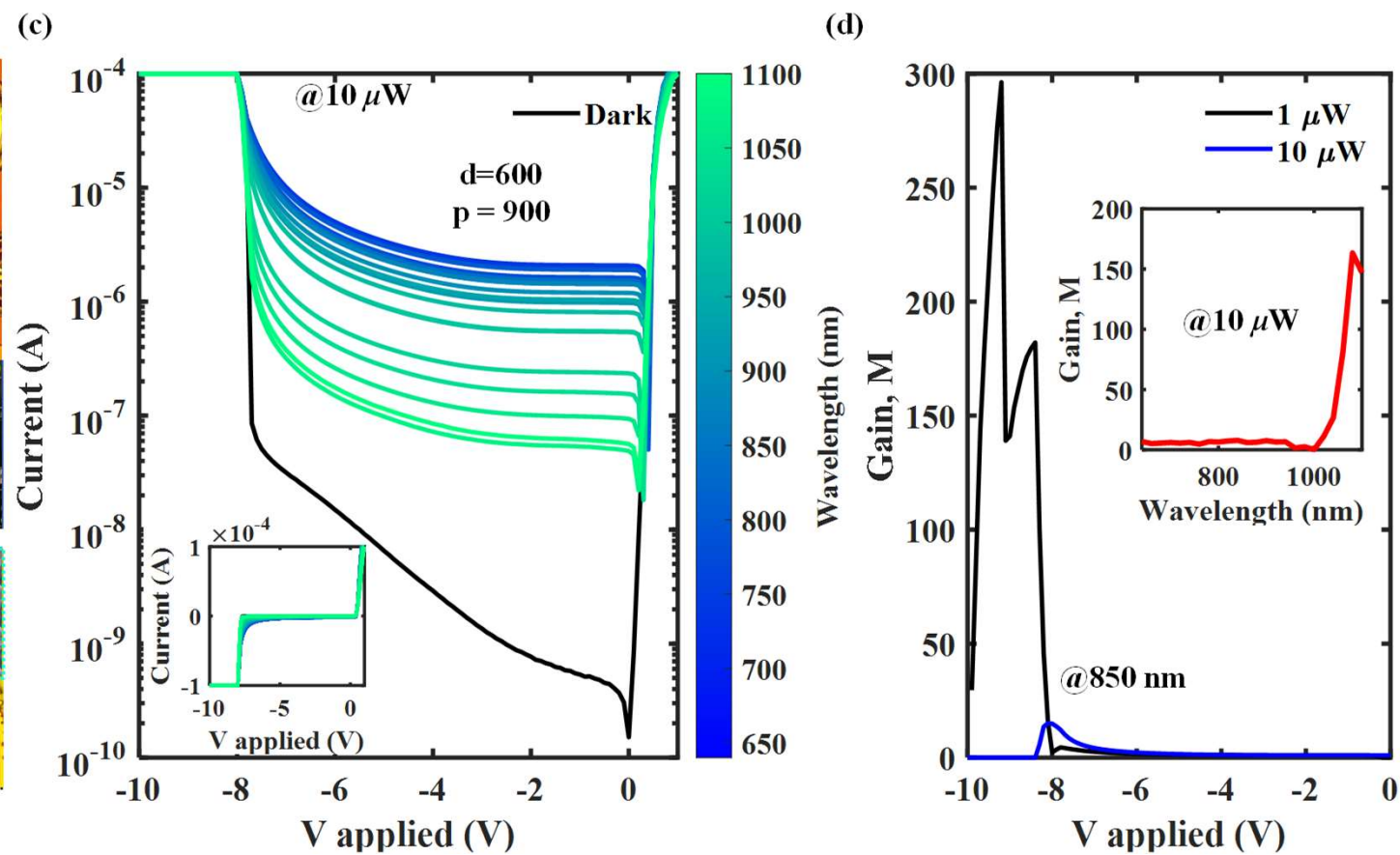

Supplement: Supplementary file 2 — ph3c00026_si_002.zip [file ph3c00026_si_002.zip › Devices_IV.pdf]

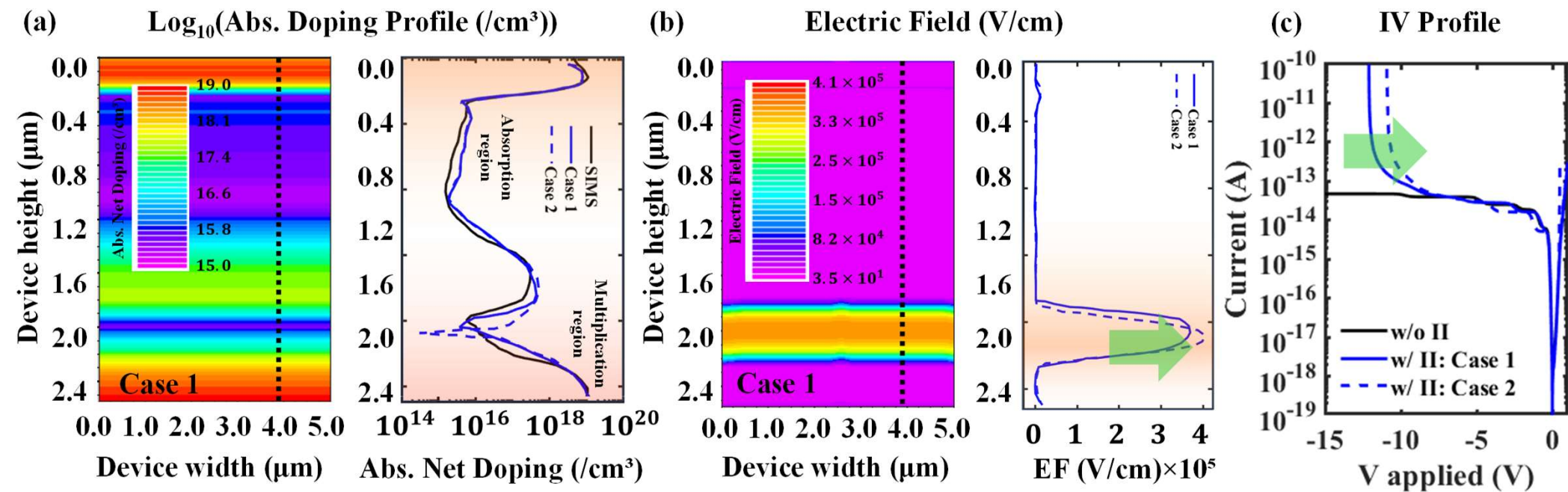

Supplement: Supplementary file 2 — ph3c00026_si_002.zip [file ph3c00026_si_002.zip › Doping_profile.pdf]

## IV Characteristics

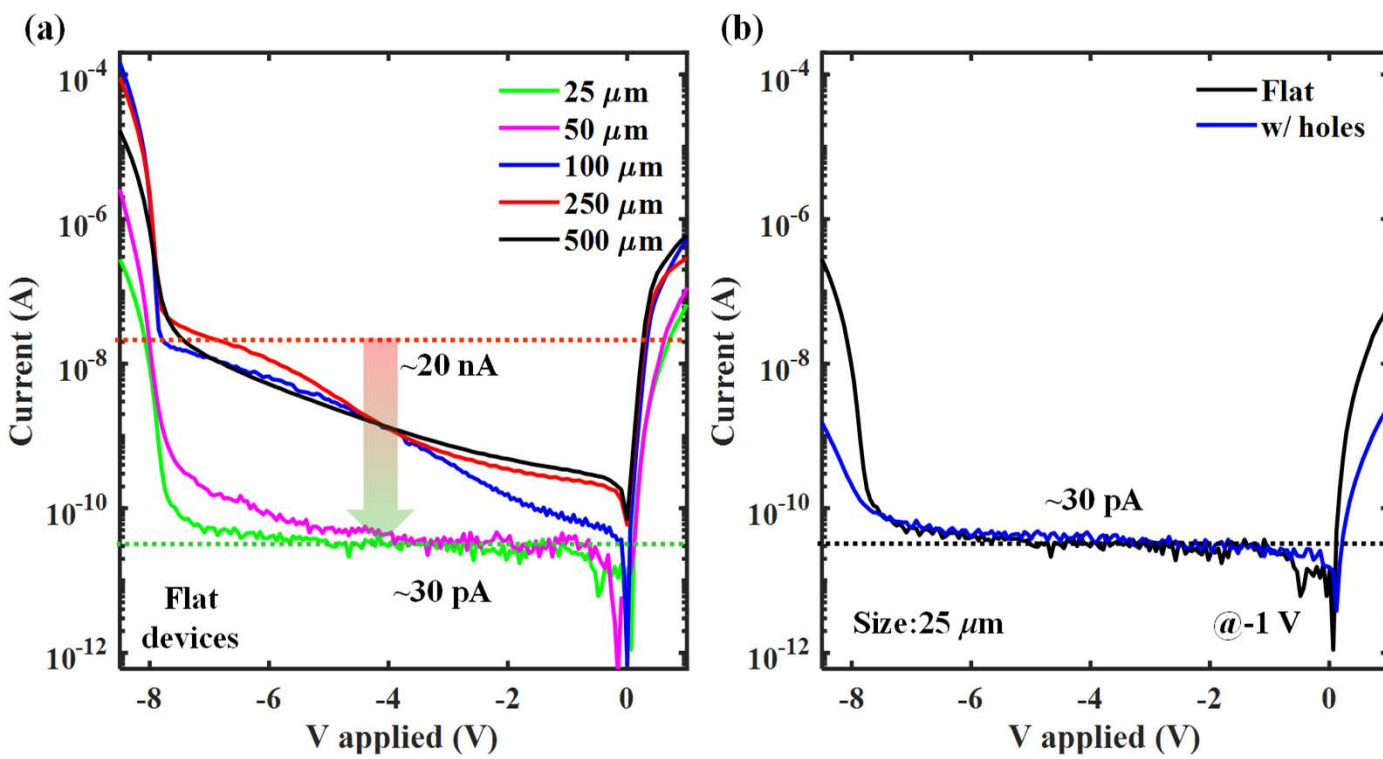

## External Quantum Efficiency

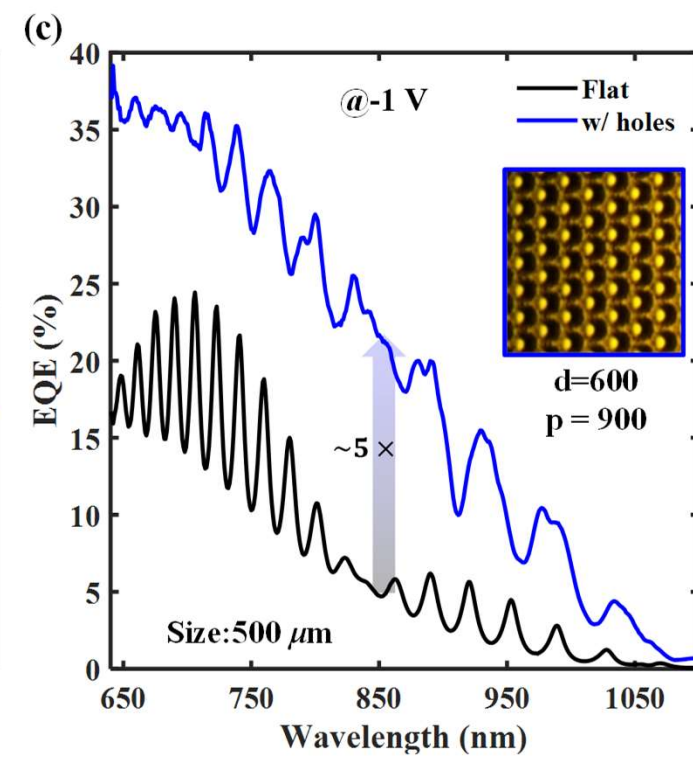

Supplement: Supplementary file 2 — ph3c00026_si_002.zip [file ph3c00026_si_002.zip › EQE.pdf]

## Si APD Fabrication process flow

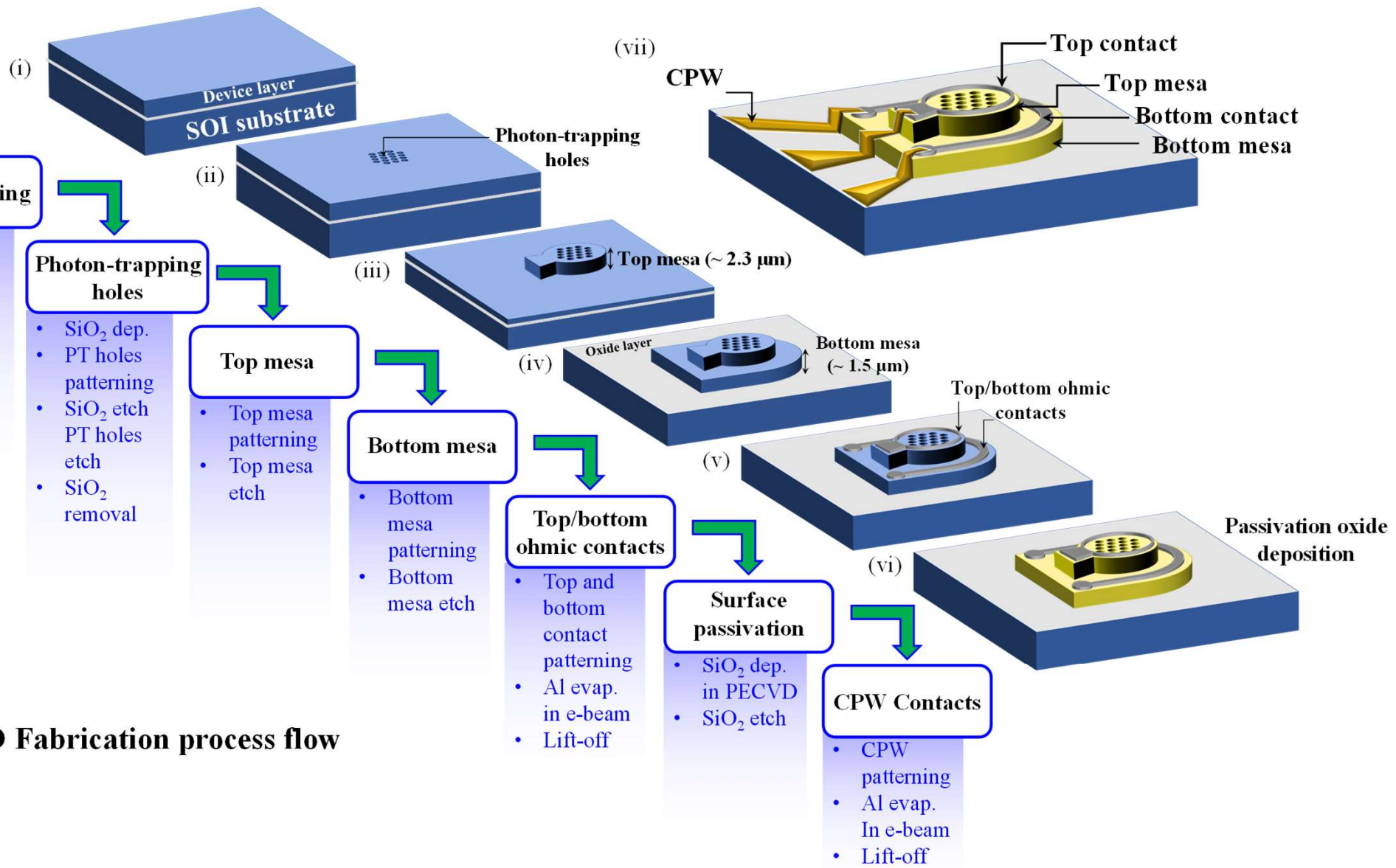

Supplement: Supplementary file 2 — ph3c00026_si_002.zip [file ph3c00026_si_002.zip › Process_flow.pdf]

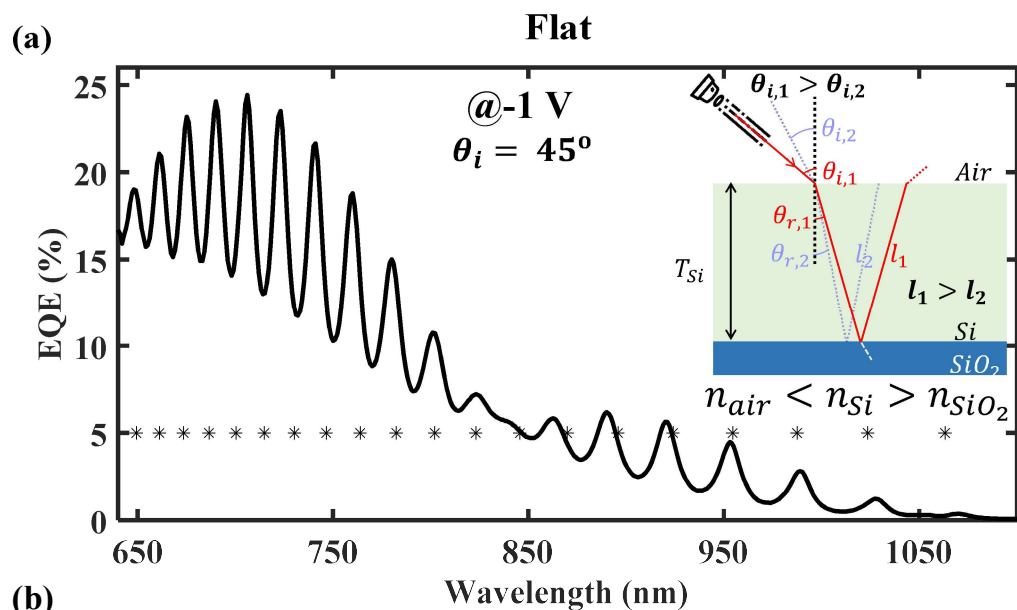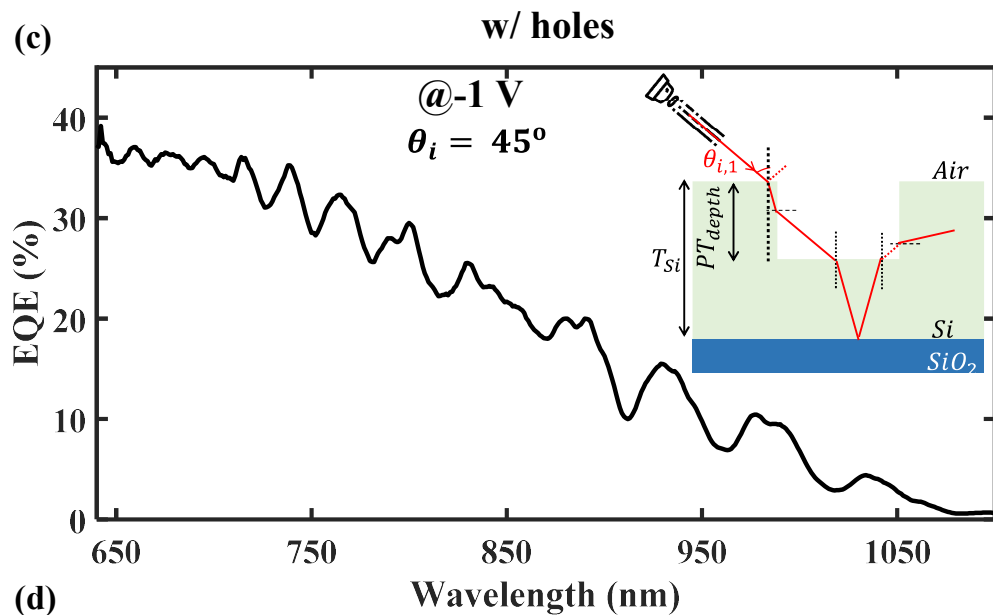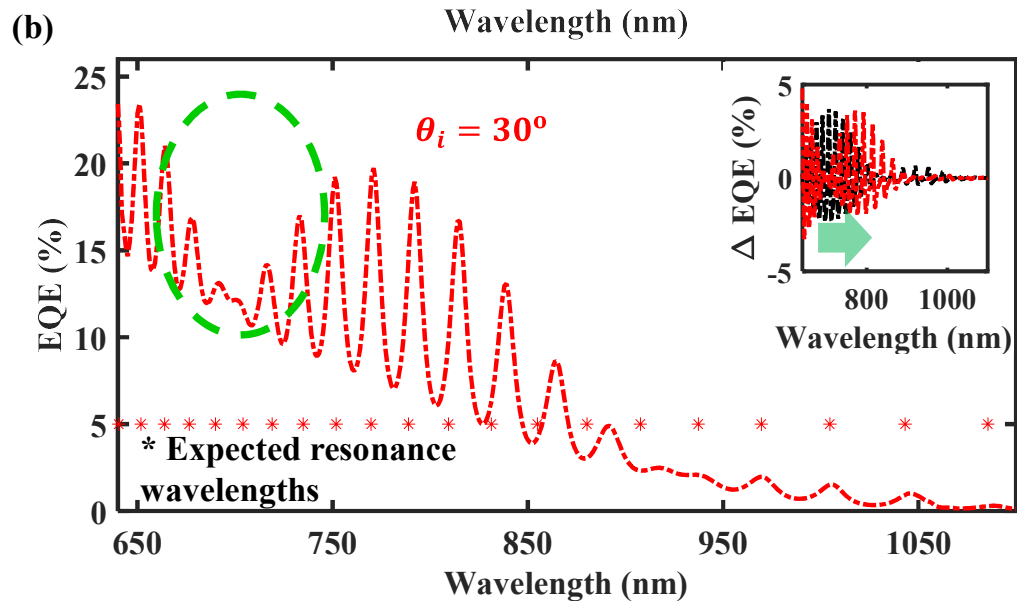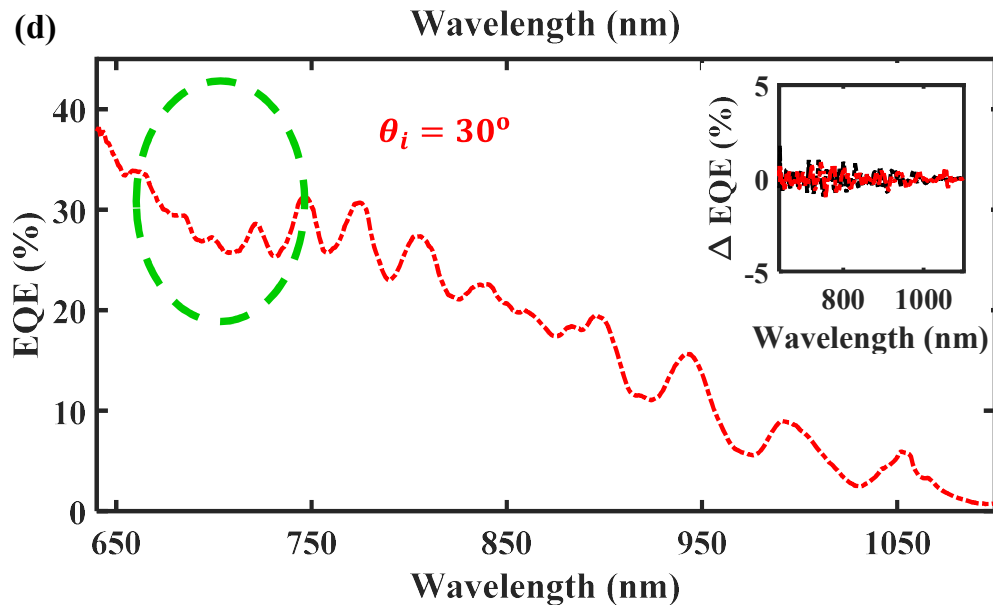

Supplement: Supplementary file 2 — ph3c00026_si_002.zip [file ph3c00026_si_002.zip › EQE_angle_dependency.pdf]

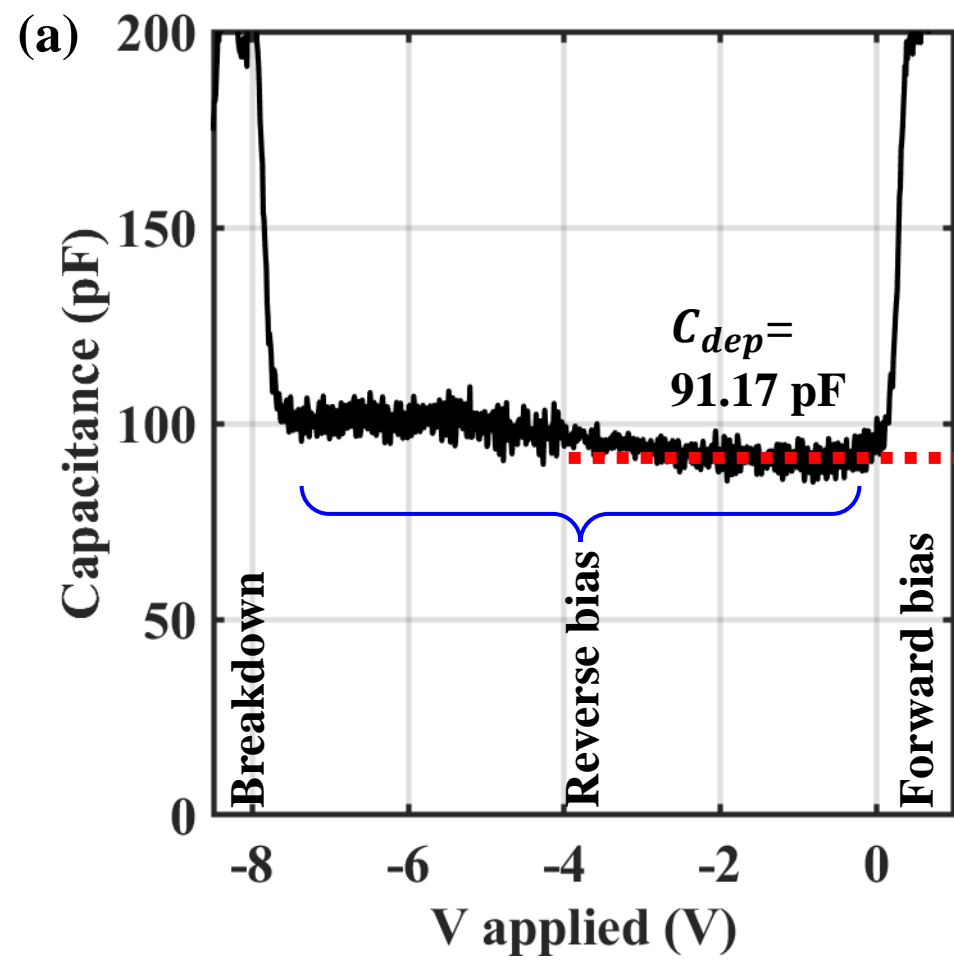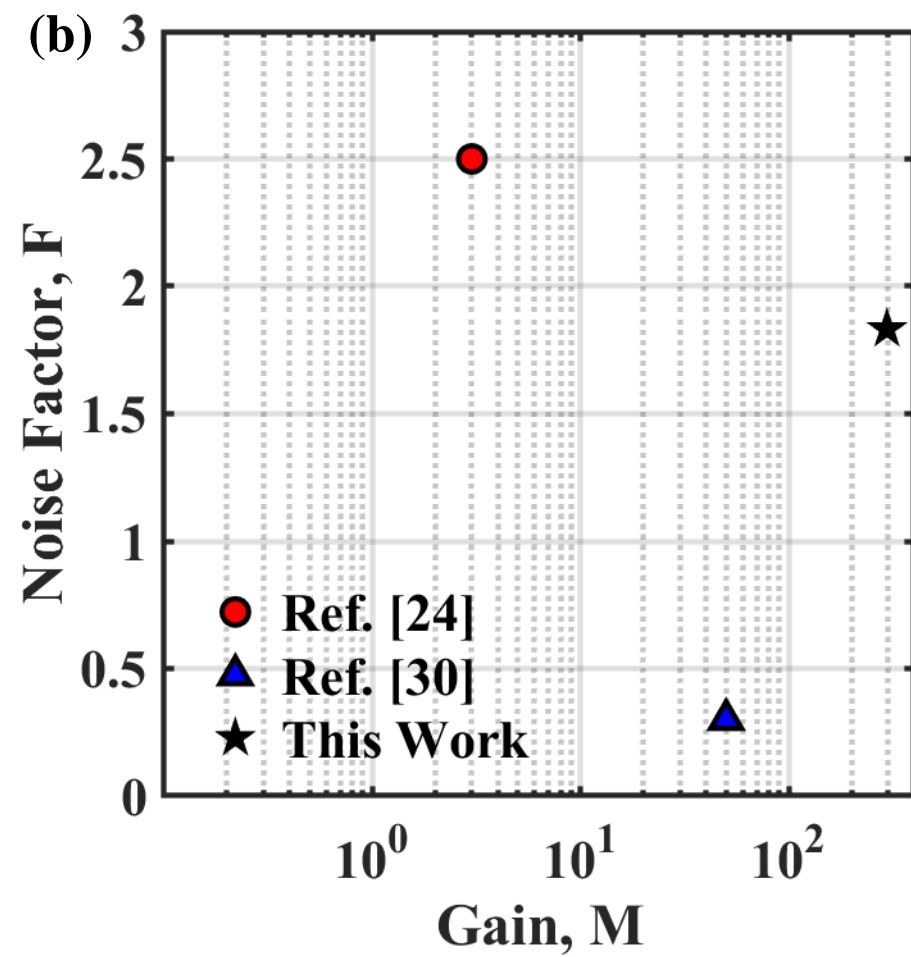

Supplement: Supplementary file 2 — ph3c00026_si_002.zip [file ph3c00026_si_002.zip › CV_and_Noise.pdf]

(a)

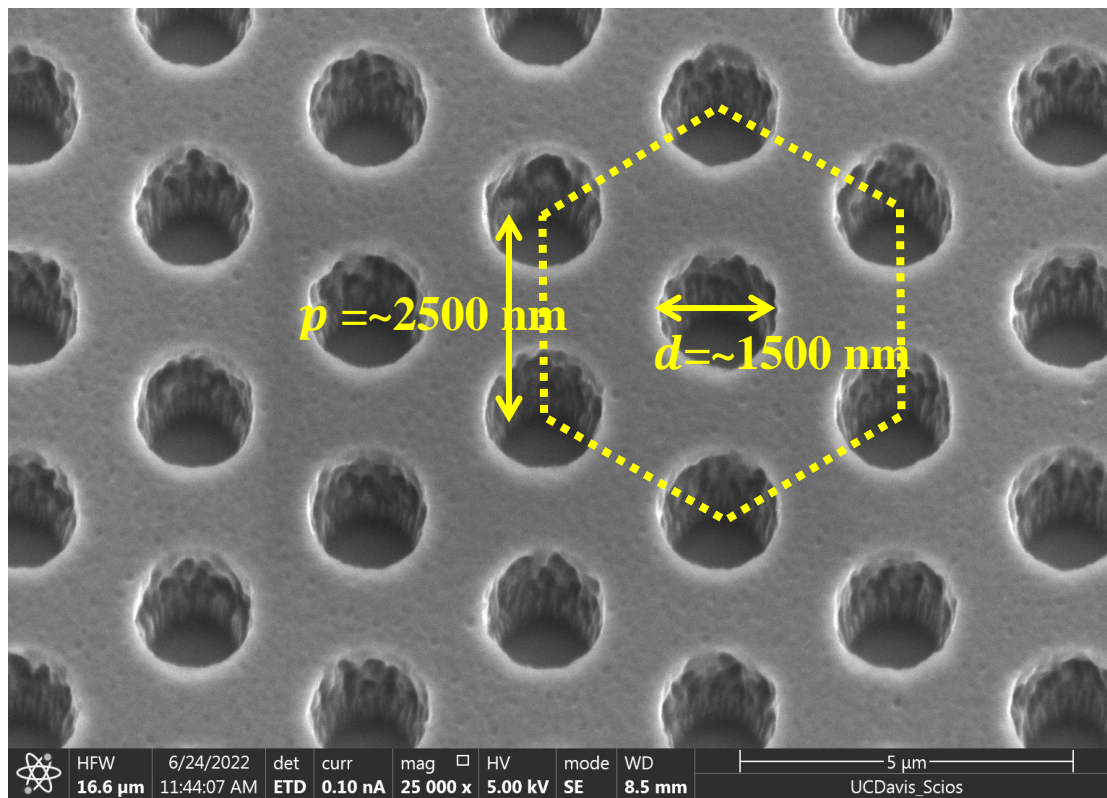

(b)

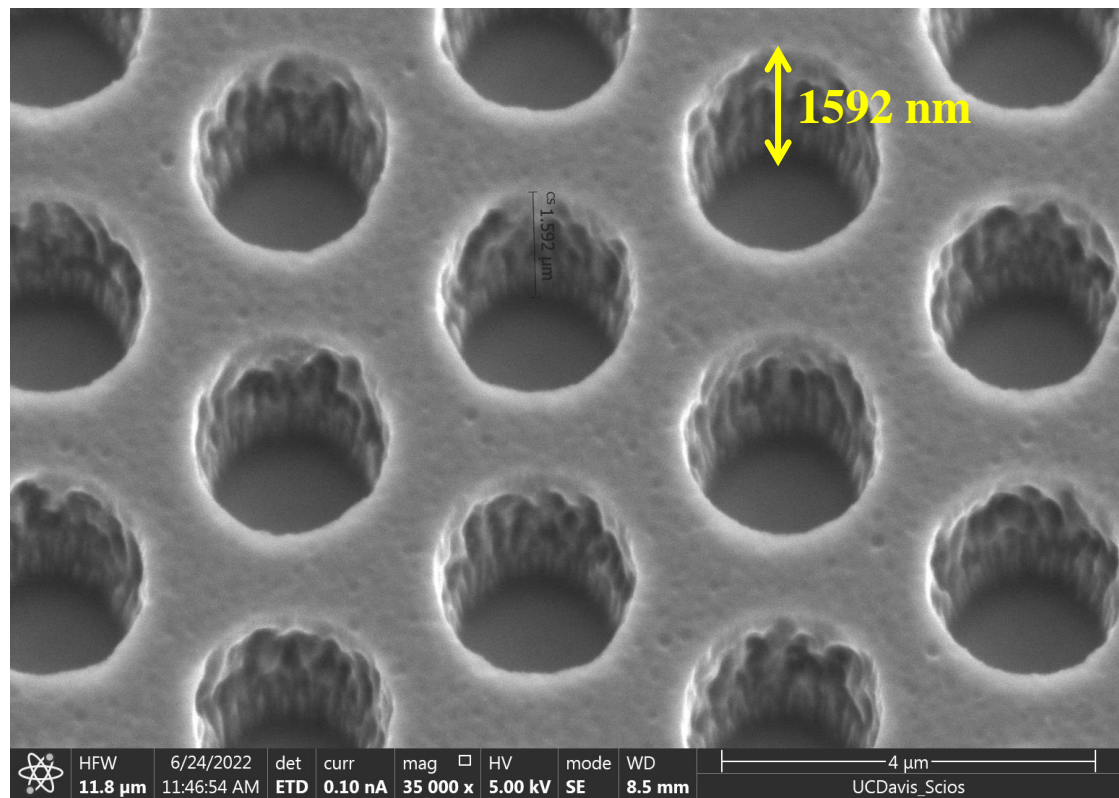

Supplement: Supplementary file 2 — ph3c00026_si_002.zip [file ph3c00026_si_002.zip › SEM_Image.pdf]
